# Cervical vasovagal shock: A rare complication of incomplete abortion case report

**DOI:** 10.1016/j.ijscr.2022.107455

**Published:** 2022-07-25

**Authors:** Willbroad Kyejo, Brenda Moshi, Vicky Kapesi, Gregory Ntiyakunze, Daud Gidion, Munawar Kaguta

**Affiliations:** a Department of Family Medicine, Aga Khan University, P.O. Box 38129, Dar Es Salaam, Tanzania; b Department of Obstetrics and Gynaecology, Aga Khan Hospital, P.O. Box 2289, Dar Es Salaam, Tanzania

**Keywords:** Cervical vasovagal shock, Incomplete abortion, Case report

## Abstract

**Introduction and importance:**

Cervical vasovagal shock is termed as stimulation either by instruments or products of conception at cervical os results into bradycardia and hypotension. In primary care settings cervical vasovagal shock can occur during insertion of an intrauterine device (IUD) or any cervical stimulation during physical examination. This case we highlight an uncommon complication of incomplete abortion which is the rare cause of cervical vasovagal shock.

**Case presentation:**

A 42-year-old Gravida 3 Para 2 Living 2 with Gestational age of 12 weeks presented with vaginal spotting for 2 days. Initial examination she was conscious with normal vital signs. However, after initiation of medical management of incomplete abortion, she had increased per vaginal bleeding with hypotension and bradycardia. Speculum examination was done; this revealed products of conceptus in cervical os and a diagnosis of cervical vasovagal shock was made. Patient was then counselled for evacuation and informed consent was sought. She was taken for evacuation; suction and gentle curettage was done. Post evacuation patients' vitals returned to normal ranges, and patient taken to the ward to continue with post procedure management.

**Clinical discussion:**

Bleeding in the first trimester is a common presentation in up to 30 % in early pregnancies and more than 50 % of those will go on to have a normal pregnancy. Most patients with incomplete abortion present at emergence department with shock, this will commonly be due to sepsis, hypovolemia, or haemorrhage. In this case report with discuss a rare cause of shock in women with incomplete abortion.

**Conclusion:**

Cervical vasovagal effect of the products of conception passing through the cervix causes a reflex bradycardia. It is crucial as physician attending women with incomplete abortion to make sure all the product of conception are passed out and in situation if there is remaining products of conception in the cervix should be removed using a sponge-holding forceps to prevent vasovagal stimulation in the cervix.

## Highlights

• Cervical vasovagal shock
• Hypotensive shock without tachycardia
• A rare complication of incomplete abortion
• Role of dilatation and curettage in cervical vasovagal shock.

## 1 Introduction and importance

Shock is a life-threatening condition of circulatory failure that most commonly presents with hypotension. It can also be heralded by other vital sign changes or the presence of elevated serum lactate levels [1], [2]. The effects of shock are initially reversible but can rapidly become irreversible, resulting in multi-organ failure (MOF) and death. Therefore, when a patient presents with undifferentiated hypotension and/or is suspected of having shock, it is important that the clinician rapidly identify the aetiology so that appropriate interventions and therapy can be administered to correct the cause of shock and prevent MOF and death [1], [2].

Cervical stimulation either by instruments or products of conception at cervical os results into bradycardia and hypotension which is referred to as “cervical shock” [3]. More commonly, in primary care settings, cervical shock can occur during insertion of an intrauterine device (IUD) [3]. The Faculty of Sexual and Reproductive Healthcare (FSRH) states that the availability of appropriate emergency medication, including atropine, during IUD insertion is essential, and this is a service standard for resuscitation in sexual health services in the United Kingdom [4].

Most cases of cervical vasovagal shock result from the presence of products of conception in the cervix during miscarriage, which should be removed as part of the treatment [4]. The main pathophysiology of cervical vasovagal shock is thought to be due to stimulation of the vagal nerve whilst uncommon, it rapidly cause a woman to become unwell with circulatory compromise [4].

This case highlights an unusual complication of incomplete abortion and the importance of making the diagnosis promptly to allow for early and appropriate management. It is important to have knowledge on cervical vasovagal shock and its causes as it varies significantly in terms of pathophysiology and management when compared to other more common causes of shock such as septic shock and hypovolemic shock which would be differential diagnoses given the patients' presentation. If this cause of shock was not promptly recognized, the patient would have received inappropriate treatment with excessive intravenous fluids and even might have decompensated further and progressed to cardiac arrest. This paper has been reported in line with the SCARE 2020 criteria [5]. This article has been registered with the Research Registry with identification number researchregistry8059 and can be found through the following hyperlink Browse the Registry - Research Registry.

## 2 Case presentation

A 42-year-old Gravida 3 Para 2 Living 2 with Gestational age of 12 weeks presented with vaginal spotting for 2 days. It was of gradual onset progressively worsening with no specific periodicity. She presented with fresh blood and one incident of passing clots, no reported products of conception seen. This was accompanied with lower abdominal pain cramping in nature, radiating to the lower back with no bowel or bladder habit change. She denied any history of nausea, vomiting or epigastric pain. She also denied any history of awareness of heartbeat, dizziness, or loss of consciousness.

Patient reported that her previous pregnancies were delivered vaginally without complication. She has no known chronic illness and denied any chronic familial illness. She denied any drug or food allergy. She has no smoking or alcohol use history.

**Fig. 1:**
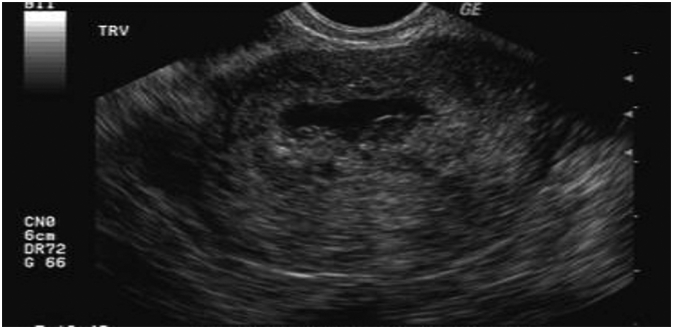
Below ultrasound showing significant retained product of conception in the uterine cavity.

On examination she was alert and oriented with a normal pulse rate of 84 beats per minute, Blood pressure 110/72 mmHg, other vital signs were normal. Abdominal examination showed normal abdominal contour, not distended, no scar, she had mild tenderness on deep palpation of the suprapubic region. Speculum examination revealed slightly open cervical os with products of conception not visible, oozing blood from external os. Systemic examination was unremarkable. A diagnosis of incomplete abortion was made, and laboratory results are as follows: White blood cell 4.6 × 10^9^/l, Hemoglobin 11.4 g/dl, Platelets 255 × 10^9^/l. *Trans*-vaginal ultrasound confirmed the findings as reported to show significant products of conception in the uterine cavity Fig. 1.

Medical management of incomplete abortion was initiated where the patient was started on sublingual misoprostol 600 microgram as initial dose. 4 h later nurse reported the patients' vitals to be 78/52 mmHg with pulse rate of 56 beats per minute. The patient reported to have increased blood clots after the dose was taken. Initial resuscitation was started with two large bore IV cannulas running 1 l of normal saline, a 14 French urinary catheter inserted that drained clear urine of 900mls, grouping and cross matching was done and requested 2 units of whole blood to be on standby. Vitals were checked 15 min later, and the patient was still deteriorating despite fluid resuscitation, peripherally shutting down and with cold extremities, blood pressure dropped to 62/40 mmHg with heart rate of 51 beats per minute. A vaginal inspection was done and revealed blood clots which where manually evacuated. A sterile speculum inspection done which revealed products of conceptus stuck at the cervical os.

Patient was the counselled for evacuation for which she consented and was taken to the operating theatre. With the patient in lithotomy position, Cusco speculum was inserted, and inspection was done. Products of conceptus were found stuck at the internal cervical os, evacuation of products from the cervical os was done using sponge holding forceps followed by gentle suction and curettage.

Soon after evacuation, the patients' vitals improved significantly to a blood pressure of 118/70 mmHg, heart rate of 65 beats per minute. The patient was reviewed 2 h post procedure in the ward and she was clinically and vitally stable where she continued with post procedure medication (Tablets Co-amoxiclav 625 mg 12 hourly for 5 days) pain medication (Tablets ibuprofen 400 mg 8 hourly for 5 days when needed), Tablets Ferrotone 1 tab daily. She was discharged on day 2 post evacuation and she was clinically and vitally stable with hemoglobin levels of 10 g/dl. She was educated on danger signs and post abortal care was done. She was told to return after two weeks for serum beta HCG levels and ultrasound scan. Post discharge the patient was faring well and her recovery was without complication.

## 3 Discussion

Bleeding in the first trimester is a common presentation in up to 30 % in early pregnancies and more than 50 % of those will go on to have a normal pregnancy [6]. However, vaginal bleeding may signify pathologies such as abortion, molar pregnancy, ectopic pregnancy and gestational trophoblastic disease [6]. The clinical manifestations of undifferentiated shock vary according to the aetiology and stage of presentation. Features that are highly suspicious for shock include hypotension, oliguria, abnormal mental status, tachypnoea, cool, clammy skin and metabolic acidosis (usually hyperlactatemia) [7]. Most clinical manifestations are neither sensitive nor specific for the aetiology of shock and are primarily used to narrow the differential diagnosis so that empirical therapy can be administered in a timely fashion [7] [8].

Patients with incomplete abortion may present to emergency department with shock, this will

commonly be due to sepsis, hypovolemia or haemorrhage [4]. Patients with an incomplete abortion and retained products of conception commonly have one or more of the following signs: uterine bleeding, pelvic pain, fever and uterine tenderness [4]. Patients with haemorrhagic shock have obvious signs of blood loss and those with septic abortion have signs of sepsis.

In our case, the hypotension and bradycardia were triggered by vagal stimulation as the products of conception passed through the cervix. As the products were removed, the vagal stimulation ceased, and the patient made a rapid recovery.

In most of the literature reviewed, patients with undifferentiated hypotension or shock airway and breathing should be stabilized with oxygen and/or mechanical ventilation, where necessary. Intravenous access should be secured so that patients can be immediately given intravenous fluids (IVF) to restore adequate tissue perfusion [9]. Resuscitative efforts should **not** be delayed for diagnostic evaluation or for central venous catheterization [9]. This is similar to the resuscitation process given to our patient before being taken to the theatre.

A study done in Cambridge by Biko et al. suggests that for a patient with symptomatic bradycardia secondary to cervical shock; cessation of cervical manipulation and removal of all instruments is advised. They also suggest the patient be kept supine with elevated legs to improve venous return. If these are efforts are insufficient to improve the patient's hemodynamic status then 500-600 μg IV atropine is recommended followed by a saline flush [10]. In our case, we identified products of conception in the cervix and consultant gynaecologist was consulted and patient was taken for curettage to prevent further complication of hypovolemic shock. Post-operative there was a complete resolution of the symptoms and patient was discharged on day 2 post procedure and her recovery was without complication.

## 4 Conclusion

Cervical vaso-vagal effect of the products of conception passing through the cervix causes a reflex bradycardia in the presence of hypotension. It is crucial that as a physician attending women with incomplete abortion to make sure all the product of conception are passed and in situation where there are retained products of conception in the cervix, there products should be removed using a sponge-holding forceps to prevent vasovagal stimulation in the cervix. It is important that all doctors working in the emergency department have knowledge of cervical vasovagal shock especially when dealing with women who present with vaginal bleeding and a positive for pregnant test.

## Patient perspectives

As I was coming to the hospital I already had a feeling I was undergoing a miscarriage but I was still hopeful maybe my baby will still be fine, but after the ultrasound results were handed over to me I was in so much pain by then, the doctors explained to me about the medical management but I was so scared to go through the process asking myself what if I bleed too much, what if the pain is unbearable but the doctors reassured me I will be under observation and I agreed to receive my first dose, shortly after I started having blood clots but my pain was well managed by pain killers, initially I didn't question myself much because I noticed the blood clots where not so significant to be frightening but shortly after I started feeling somehow lightheaded, there was a lot of haziness, I felt as if I was going to faint so soon despite that I was already lying down, doctors rushed and there was so much going on, nurses and doctors trying to solve what was wrong I became so scared, then was rushed to theatre where I don't remember much, but I felt so much better after coming back to the ward from theatre, I even thought I would probably going to a need a blood transfusion after the procedure but doctors assured me, I'm doing just fine that all I need is some few medications and I will be good to go home the next day.

## Sources of funding

No funding was provided for research.

## Author contribution

W.K: Study conception, production of initial manuscript, collection of data.

B.M: Production of initial manuscript, revision of the manuscript, proofreading.

V.K: Revision of the manuscript, proofreading.

D.G: Production of initial manuscript, collection of data.

G.N: Revision of the manuscript, proofreading.

M.K: Study conception, revision of the manuscript, proofreading.

## Guarantor

Dr. Munawar Kaguta, Obstetric and Gynaecologist, Aga Khan Hospital.

## Provenance and peer review

Not commissioned, externally peer-reviewed.

## Consent

Written informed consent was obtained from the patient for publication of this case report and accompanying images. A copy of the written consent is available for review by the Editor-in-Chief of this journal on request.

## Registration of research studies

researchregistry8059.

## Guarantor

Dr. Munawar Kaguta.

## Declaration of competing interest

No conflicts of interest.
